# Machine Learning-Empowered Real-Time Acoustic Trapping: An Enabling Technique for Increasing MRI-Guided Microbubble Accumulation

**DOI:** 10.3390/s24196342

**Published:** 2024-09-30

**Authors:** Mengjie Wu, Wentao Liao

**Affiliations:** 1 Department of Mechanical Engineering, The University of Hong Kong, Hong Kong 999077, China; 2 Medical Imaging Center, Shenzhen Hospital of Southern Medical University, Shenzhen 518005, China; liaowentao628@i.smu.edu.cn

**Keywords:** acoustic trap, microbubble (MB), machine learning, magnetic resonance imaging (MRI), heterogeneous media

## Abstract

Acoustic trap, using ultrasound interference to ensnare bioparticles, has emerged as a versatile tool for life sciences due to its non-invasive nature. Bolstered by magnetic resonance imaging’s advances in sensing acoustic interference and tracking drug carriers (e.g., microbubble), acoustic trap holds promise for increasing MRI-guided microbubbles (MBs) accumulation in target microvessels, improving drug carrier concentration. However, accurate trap generation remains challenging due to complex ultrasound propagation in tissues. Moreover, the MBs’ short lifetime demands high computation efficiency for trap position adjustments based on real-time MRI-guided carrier monitoring. To this end, we propose a machine learning-based model to modulate the transducer array. Our model delivers accurate prediction of both time-of-flight (ToF) and pressure amplitude, achieving low average prediction errors for ToF (−0.45 µs to 0.67 µs, with only a few isolated outliers) and amplitude (−0.34% to 1.75%). Compared with the existing methods, our model enables rapid prediction (<10 ms), achieving a four-order of magnitude improvement in computational efficiency. Validation results based on different transducer sizes and penetration depths support the model’s adaptability and potential for future ultrasound treatments.

## 1. Introduction

Harnessing acoustic interference within the human body, ultrasound has expanded its applications beyond diagnostic imaging to therapy. Focused ultrasound (FUS) is a typical non-invasive treatment that relies on acoustic interference to generate a focal spot, successfully inducing evident temperature rise for thermal ablation inside organs [1], such as the brain [2], liver [3], and uterus [4]. It can also vibrate target tissues to stimulate mechanical effects in non-thermal applications, among which FUS-mediated blood–brain barrier opening has advanced to clinical Phase II [5]. In addition, inspired by optical tweezers, FUS has also been employed to capture microparticles [6]. This phenomenon arises from the acoustic radiation force (ARF) exerted by the focused ultrasound waves, which creates a stable aggregate at the focal spot [6]. Apart from enabling waves to be focused at a point, acoustic interference can form a “well”-shaped acoustic pressure zone [7,8]. Within the zone, exterior high pressure is converged towards the interior with the lowest amplitude [9,10]. This pressure gradient results in ARF, such that this zone can highly localize solid objects with a diameter of sub-wavelength (<λ) [11], e.g., cells (Ø1 μm–30 μm [12,13]) and bacteria cluster (Ø45.6 μm–228 μm [14]). Therefore, both the focal beam and “well”-shaped pressure zone are generally referred to as “acoustic trap” [7,9,10]. In the latest decade, some laboratory investigations have showcased ARF is sufficient to withstand object trapping against the low-velocity (e.g., 2.5 mm/s) flow in water tanks [11,15]. More recently, the trap successfully located MBs and thereby increased its aggregation in mouse capillaries, showing its applicability for *in vivo* applications [16]. Accredited to these aforementioned advances, the acoustic trap deserves further exploration for the treatment of vascular-rich solid tumors, e.g., hepatocellular carcinoma (HCC) [11,15]. In current preclinical practice, the FDA-approved ultrasound-mediated MBs (Ø1–10 μm [16]) successfully increased local anti-tumor drug (e.g., Doxil) accumulation because the induced microbubble oscillation increases permeation of membranes and enhances drug diffusivity [17,18]. However, the drag forces generated by high-velocity pulsatile (1–4 cm/s [16]) in feeding microvessels limit the MB retention time, leading to heavy drug leakage and reduced absorption [16]. Although optical tweezer [19,20,21], dielectrophoresis [22], and electrokinetic tweezer [23] have achieved contactless trapping of biomolecules, optical beams are unsuitable for use in opaque medium, and the current of electrokinetic tweezer and dielectrophoresis can induce heating, thereby affecting cell physiology [16]. By contrast, ultrasound waves can penetrate thick tissues and possess excellent biocompatibility [24]. Therefore, the feasibility of acoustic trap generation within human organs, potentially prolonging the retention of MBs in counterflow [16], is highly desired.

**Figure 1:**
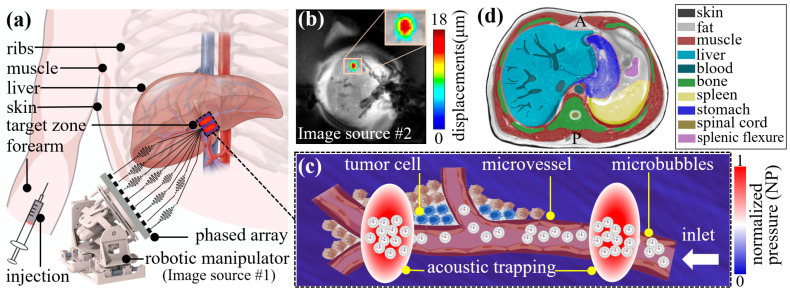
Overview of trapping drug carriers using acoustic trapping. (**a**) Acoustic trap generation within human liver using phased array. MR-compatible robotic manipulator (Image source #1) [27] positions the array towards the liver. The emitted beams pass through tissues (e.g., skin, fat, muscle, and ribs) to generate a trap at the given target zone. MBs are injected via radial artery of the forearm [45]. (**b**) Example of focal spot visualization in *ex vivo* porcine kidney via MR-ARFI (Image source #2 [29]). (**c**) Close-up illustration of MB accumulation in a microvessel due to acoustic trapping. Two finger-like high-pressure (warmer color) regions locate MBs around the tumor cells. (**d**) Segmentation of MR T2 image for FE modeling in both data acquisition and performance validation process. Letters “A” and “P” in black color indicate the anterior and posterior abdominal walls, respectively.

This envisioned medical potential of acoustic trapping is tightly bound to the capability of tracking carriers and sensing ultrasound interference. It not only provides spatial guidance for payload release but also monitors if the trapping beams are maintained on the target zone (Figure 1a) [18]. Currently, magnetic resonance imaging (MRI) has enabled real-time carrier tracking in *in vivo* trials attributed to magnetic resonance (MR) imageable materials [25]. Apart from carrier tracking, MRI can sense ultrasound interference by measuring resulting temperature changes or tissue displacements [26]. Specifically, MR thermometry has achieved real-time heat deposition monitor to sense focal spots in FUS [26,27]. Recently, MR acoustic radiation force imaging (MR-ARFI) emerged as an alternative to MR thermometry, addressing imaging accuracy degradation in tumors surrounded by fat via detecting micro-scale tissue displacement (Figure 1b) [28,29,30]. These advances show that MRI-guided MB tracking and trap sensing are becoming immensely promising for medical scenarios. Nevertheless, the accurate and rapid trap generation at the target remains a formidable challenge, as the heterogeneous tissues complicate the computation of beam propagation [31]. Specifically, as shown in Figure 1a, ultrasound undergoes numerous refractions between tissue layers (e.g., fat, muscle, and bone) with varying thicknesses, resulting in a highly curved propagation pathway within the body. Then, considering that each pathway has its unique pressure attenuation coefficient, the beams emitted by multiple transducers exhibit a non-uniform wavefront when arriving at the target. This would weaken the intensity profiles of acoustic traps. Moreover, variations in body tissue structure would induce phase distortion to the propagating waves, thus decreasing the computation accuracy. Furthermore, the computational efficiency becomes an aggravated challenge when fine adjustment to trap position is required to target a more suitable MB seeding site or microvessel, relying on the MRI-guided MB monitor. The short circulation lifetime of most MBs (typically 3–5 min [32]) underscores the urgency of generating a new trap before MBs rupture.

Tracing back the progress in trap generation, researchers primarily focused on hardware and algorithm design. For hardware, the single-side phased array is increasingly favored [33,34]. Compared to the dual-sided [35,36], closed [37,38], or spiral [39] array arrangement, it eliminates spatial constraint so as to fit the parts of the body [33]. Assisted by cumbersome mechanical actuation, the system can relocate the trap in two dimensions (2D) or three dimensions (3D). Yet, such an approach offers restricted dexterity for fine-tuning trap positions [34,35]. Then, a few algorithms for electronically steering elements’ phase and amplitude have been developed, permitting the generation of dynamic traps without mechanical actuation [10,14]. Phase is the most critical variable, determining the trap generation and its position [10]. Amplitude attenuates during propagation due to absorption, refraction, etc., influencing the trap’s acoustic intensity profiles [15]. Among the existing algorithms, only three, namely, iterative backpropagation (IB), time reversal-based method (TRM), and holographic acoustic element framework (HAEF), have been applied in heterogeneous media [10,14,40]. IB, introduced in 2019 [41], only enabled trap generation in a mouse’s skin-fold chamber model (tissue thickness: <1 mm) and cannot solve the complicated acoustic field [14,42]. Its application to thick tissues, phantoms, or the human body remains unachieved. In contrast, TRM has been repeatedly used in large-volume tissues [43]. It converts phase calculation into computing the beam’s propagating time between elements and target in the time domain, termed time-of-flight (ToF). Owing to TRM’s inherent capability of correcting phase distortion, it can achieve accurate solutions. Moreover, TRM can compute the amplitudes, thus allowing the algorithms to steer elements’ emission pressure to correct the non-uniform wavefront. However, TRM primarily relies on numerical computation and still has low efficiency for ToF and amplitude computation. For instance, Yang et al. used the k-Wave toolbox to compute ToF as beams traversed through a macaque skull (65 mm × 68 mm × 38 mm) [43]. This method usually costs an excessive computational expense (e.g., tens of minutes for per target). Regarding HAEF, it diversifies the types of traps for enhanced maneuverability [40], such as twin, vortex, and bottle traps [10], beyond the conventional focal beam created by IB and TRM. To date, HAEF’s iterative phase computation is quite time-consuming and faces challenges in mathematically converging [42]. Additionally, the tissues inevitably induce phase distortion to the beams and weaken the accuracy of phase computation. To seek precision, Cao et al. positioned a hydrophone at the target for phase calibration and produced a twin trap after waves passed through a porcine rib [40], but this device is not suitable for non-invasive treatments. Worse yet, HAEF does not compute the pressure amplitude, which means it cannot correct the non-uniform wavefront. Thereby, the weakened acoustic intensity profiles in tissues cannot be improved by amplitude modulation (AM) [44]. To sum up, these methods still present substantial limitations for medical potential.

To advance the medical potential of acoustic traps for increasing MRI-guided MB accumulation, we propose a future-proof technique that leverages machine learning to deliver trap generation in heterogeneous media. As shown in Figure 1c, the acoustic trap located at the predefined microvessel will accumulate the MBs and counteract the blood pulsatile, thereby potentially prolonging the retention time of MBs. The 2D segmented MR slices contain various tissue materials, such as skin, fat, muscle, liver, and bone, acting as heterogeneous media (Figure 1d). Considering the computation inefficiency of both TRM and HAEF, machine learning is expected to rapidly predict key parameters of beam propagation. Recently, a learning-based model has validated its efficacy and efficiency (~200 ms) for holographical phase modulation (PM) in a single medium [42]. To date, learning-based PM in heterogeneous media has not been studied before. Therefore, the proposed learning-based model is designed to predict beams’ ToF and amplitude in 2D heterogeneous media, accommodating multiple trap types (e.g., focal beam and twin trap). The predicted amplitude will be applied to modulate elements’ emission pressure, thereby correcting the non-uniform wavefront. To this end, two artificial neural networks (ANN) are used to create the inverse mapping from the given trap position to elements’ actuation signals (i.e., ToF and amplitude, respectively). These two ANNs can achieve high computation efficiency (<10 ms per target) much faster than the existing methods (e.g., TRM). Presently, as a pioneering work on learning-empowered trap generation, we collect the training dataset via 2D finite element (FE) modeling. The key work contributions are differentiated as follows:

(1) Development of the first machine learning-empowered model to facilitate the generation of acoustic traps in 2D heterogeneous media. This approach delivers accurate generation of multiple types of traps (e.g., focal beam and twin trap).
(2) High computation efficiency for enabling rapid phase-amplitude (PA) modulation on transducer array. The model can predict time-of-flight (ToF) and pressure amplitude within 10 ms to modulate all elements, which is significantly faster (four orders of magnitude) than the existing method (e.g., TRM).
(3) FE-based validations on the prediction performance using MR images, based on three factors, i.e., sample density, element diameters, and penetration depths, supporting our model’s potential for medical applications. FE modeling further demonstrates the capacity of twin traps for trapping microbubbles (negative acoustic contrast factor).

## 2. Materials and Methods

This section details the learning-based method dedicated to acoustic trap generation. We provide comprehensive information for model architecture, dataset collection, model training, and validation. To facilitate the reproducibility of our work, all designed files are accessible on the GitHub repository (https://github.com/mengjwu/acoustictrap, accessed on 6 September 2024), such as FE modeling files, MR anatomy models, and Python codes.

### 2.1. Learning-Based Trap Generation Model

**Figure 2:**
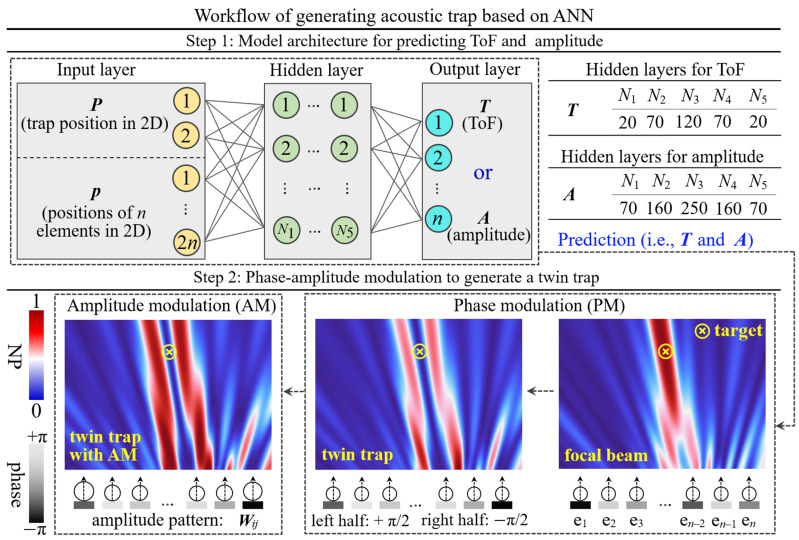
ANN-based workflow for acoustic trap generation. Step 1 illustrates the architecture of a learning-based model for predicting ToF or amplitude. The variable *n* is the element number. The proposed two ANNs have 2*n* + 2 input variables, five hidden layers and *n* outputs but diverge in hidden layers’ node layout (i.e., *N*_1_, *N*_2_, …, *N*_5_) for ***T*** and ***A*** prediction. Step 2 depicts the phase–amplitude modulation process applied to *n* elements (e_1_, e_2_, …, e*_n_*), including phase modulation (PA) and amplitude modulation (AM). Circles’ radii are proportional to the element’s emission pressure, and the grey scale of elements represents the phase pattern. A blue–red color bar is used to characterize the normalized pressure (NP) across all acoustic fields.

An acoustic trap is expected to be formed at a predefined target position; therefore, elements’ actuation signals, i.e., phase and amplitude, will have to be modulated rapidly and accurately. By TRM, the phase can be directly converted from ToF (Equations (3) and (4)). Thus, the proposed learning-based model aims to create the inverse mapping from the prescribed trap position to ToF and amplitude. As shown in Figure 2, the trap position and the elements’ position are the input variables, denoted as $P\;\in \;{\mathbb{R}}^{2}$ and $P\;\in \;{\mathbb{R}}^{2n}$, respectively, where *n* is the element number. Two ANNs with *n* output nodes were designed to predict ToF ($T\;\in \;{\mathbb{R}}^{n}$) and amplitude ($A\;\in \;{\mathbb{R}}^{n}$), respectively. Note that they feature different node layouts in hidden layers. Five hidden layers’ node numbers are denoted as *N*_1_, *N*_2_, …, *N*_5_.

Upon finalizing the prediction, phase–amplitude modulation to the phased array was performed as illustrated in step 2. ***T*** was used for computing the focal beam’s phase patterns $H\;\in \;{\mathbb{R}}^{n}$ (Equation (3)), and ***A*** was for computing the amplitude pattern $W\;\in \;{\mathbb{R}}^{n}$ (Equation (5)). Specifically, a time reversal process was conducted in PM, which converts ToF to phase for generating the phase patterns (***H***) of focal beam [11,43]. Following HAEF [10], ***H*** acts as an acoustic lens that focuses beams at target, functioning as the “single-beam trap” to ensnare objects [6,14]. Then, by adding a fixed holographic signature, the focal beam can be transformed into diverse acoustic structures, e.g., vortex, bottle, and twin [10]. For twin trap, the signature (i.e., π-phase difference) can be interpreted as a phase shift of +π/2 and −π/2, and they are added to the left and right half of array, respectively, yielding phase patterns of the twin trap (Equation (4)), denoted as $M\;\in \;{\mathbb{R}}^{n}$. Due to the different attenuation levels across all transmission pathways, the wavefront arriving at the target cannot remain uniform. As a result, the acoustic intensity profiles become weakened, such as the lower focusing quality in single-beam trap. Therefore, we introduced the amplitude patterns ***W***, consisting of *n* coefficients, to modulate element’s emission pressure, aiming to recreate a uniform wavefront and optimize the trap’s intensity profiles.

### 2.2. FE-Based Dataset Collection

**Figure 3:**
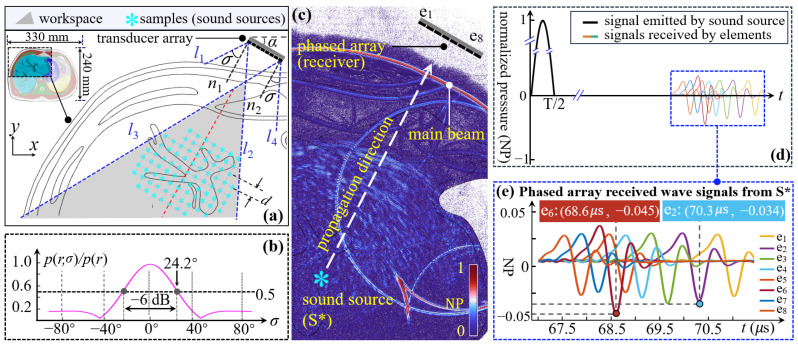
Overview of FE-based data collection process. (**a**) Planning of array position and workspace for data collection. An MR T2 image (330 mm × 240 mm) was segmented to build a 2D geometry model for FE modeling. Boundaries *l*_2_ and *l*_3_ outlined the workspace, and these samples were distributed around the vessel with a spacing of *d*. (**b**) Pressure attenuation in relation to spread angle (2σ). At the critical angle of σ = 24.2°, the pressure attenuates by half (−6 dB) over the same propagating distance *r*. (**c**) Simulated acoustic field in COMSOL. Eight elements acted as receivers to capture wave signals from one representative sound source S* (**d**) Signal emitted by sound source S* and received signals by the array. (**e**) Close-up illustration of the received wave signals. Eight peak amplitudes and their timestamps denote, respectively, ***A*** and ***T***.

To train the proposed trapping modulation model, dataset will have to be collected, consisting of the target positions, elements’ positions, ToF, and amplitude. ToF and amplitude were computed using Pressure Acoustics, Transient module from COMSOL Multiphysics. FE-based experiments have demonstrated considerable accuracy for acoustic trapping, affirming its reliability as a ground truth [46]. To ensure our simulation accuracy, the maximum mesh size was set at 1/8 wavelength [47]. The data collection was conducted based on the time reversal principle, as it can correct the phase and amplitude distortion [44]. The detailed steps were as follows: (1) A point (Øλ/20) was positioned at the target to emit waves, implying that each sample point represents a sound source, as shown in Figure 3a; (2) Elements acted as receivers to capture ToF and amplitudes from their received signals. Given the reciprocity of the beam transmission pathway, elements retransmit waveforms in the time-reversed order of ToF, allowing these waveforms to be refocused at the position of initial sound source [43].

We aimed to produce acoustic traps within the liver anatomy; thus, an open-access accessible abdominal MR image [48] was manually segmented to act as the geometry of FE modeling. The array was placed near the subxiphoid to reduce ribs’ disturbance (e.g., 10–20 times higher absorption than soft tissues [18]) [49]. In Figure 3a, the predefined targets are situated around a vessel so that the formed trap would be located at its branching microvessels. Each column of samples is parallel to normal line (e.g., *n*_1_). Moreover, referring to parameters outlined in Table 1, our elements have a diameter of 3.7 mm and operate at a frequency of 1.0 MHz. To increase the efficiency of energy delivery to the target region, the array plane should be parallel to the vessel by adjusting angle α (α = 30° in our study). Some MR-compatible robots, embedded with MR-based positional sensing markers, can be used to facilitate the precise adjustment of array pose [27,50,51]. Upon finalizing the array’s position and orientation, we outlined the effective workspace using two boundaries *l*_2_ and *l*_3_ for dataset collection. The boundaries were determined by the beam spread angle (2σ), which defines the −6 dB pressure attenuation over an identical propagating distance *r*, comparing the pressure p(r,σ) at the boundary to pressure p(r) along the normal line [52]:

(1) $$p(r,\sigma )=p(r)\frac{2J_{1}(ke\sin(\sigma ))}{ke\sin(\sigma )}$$

where $J_{1}(ke\sin(\sigma ))$ is the Bessel function of first order; *k* (=2π/λ) is the wave number; *e* is the element radius. As shown in Figure 3b, the critical attenuation of −6 dB corresponds to the angle of σ= 24.2°. After outlining the workspace, we introduced the sample density (*D*), defined as the reciprocal of the spacing (*d*) between two adjacent samples, and conducted the data collection at *D* = 1/λ. Figure 3d shows a half cycle of sinusoidal wave emitted by the sound source (S*) and the signals received by eight elements. Figure 3e displays the close-up illustration of the received wave signals, in which eight peak amplitudes and their timestamps represent amplitude and ToF, respectively. All FE modeling was conducted on a processing computer (Ryzen Threadripper 3990X, 32 GB RAM, AMD, USA).

### 2.3. Model Training

After the data collection, the model needs to be trained to predict the elements’ actuation signals so as to generate an acoustic trap at the target. All training and prediction were conducted on a notebook with an Intel i5-7360U CPU and 8 GB RAM. A total of *m* pairs of samples were acquired, and each pair included ***P***, ***p***, ***T***, and ***A***. A total of 74.5%, 13%, and 12.5% of samples were used for model training, validation, and hold-out testing, respectively. We implemented the model using Scikit-learn with L2 loss (*L*)

(2) $$L=\frac{1}{m}{\sum_{i=1}^{m}\left||x_{i}-{x_{i}}^{*}|\right|}_{2}$$

where $x_{i}$ represents the predicted values and ${x_{i}}^{*}$ represents the labeled values; *i* = 1, 2, …, *m* is the index of samples. ReLU was selected as the activation function. The trained model has 18 input variables. Five hidden layers feature a total of 300 nodes for ***T*** prediction and 720 nodes for ***A*** prediction (Figure 2). Then, the prediction results were applied to phase–amplitude modulation on array.

### 2.4. FE-Based Trap Visualization

FE modeling is a typical method to validate whether the modulated acoustic traps/HIFU align with the target [15,54]. We used COMSOL Multiphysics to visualize the acoustic field pattern based on the predicted results. Eight elements were activated via phase–amplitude modulation following the four-step process outlined below: (1) computing the maximum ToF (***T***_max_) and the maximum amplitude (***A***_max_) of the eight beams originating from the same sound source; (2) converting ToF to focal beam’s phase patterns based on time-reversed order. The phase patterns are expressed as:

(3) $$H_{ij}=\frac{2\pi (T_{\max}-T_{ij})}{T}$$

where T (=1 µs in this study) is the period of ultrasound beams; (3) and then adding the holographic signature to form the twin trap’s phase patterns. The twin trap’s phase patterns are expressed as:

(4) $$M_{ij}=H_{ij}+\frac{\pi }{2}-\pi \cdot \mathrm{Heaviside}(j-\frac{n+1}{2})$$

(4) The amplitude modulation patterns were calculated (***W***) as follows:

(5) $$W_{ij}\cdot A_{ij}=A_{\max}$$

These coefficients modulate the elements’ emission pressure based on their respective attenuation levels along the transmission pathways, allowing all beams to contribute equal pressure to the focal beam. Through the phase–amplitude modulation, the element’s actuation signals $\Psi_{ij}$ for focal beam generation are expressed as:

(6) $$\Psi_{ij}=\vartheta W_{ij}\cdot \exp(j\cdot H_{ij})$$

where j is the imaginary number and ϑ is a constant. This constant is set at 1000, indicating that the default emission pressure of elements is 1000 pascals. This setting permits modification, enabling linear scaling of the entire field pressure. Then, signals $\Psi_{ij}$ were imported to the Pressure Acoustics, Frequency Domain module from COMSOL to actuate transducer elements.

### 2.5. FE-Based Microbubbles Trapping

We used numerical simulations to display the MB trapping process in a twin trap. A simple 2D model was set up in COMSOL, which included the trap’s ARF, gravity, and drag force of fluid. ARF was produced by eight modulated transducers, and the maximum pressure of the acoustic field was about 340 KPa [55]. Drag force was exerted by a pulsatile flow with a period of one second. The pulsatile’ inflow velocity was expressed as signal *Pul*(*t*):

*Pul*(*t*) = 13.83 × *vmin* + 1.383 × (*vmax − vmin*) × (3sin(2πt)^2^ + abs(sin(2πt)) × sin(2πt) + sin(πt)^2^ + 42 × (sin(2πt) + abs(sin(2πt))) × exp(−20 × (t − round(t − 0.5))) × 1.35/(1 + exp(130t + 10)))(7)

where *vmin* (=0.5 mm/s in our work) is the minimum inflow velocity and *vmax* (=1.54 mm/s in our work) is the maximum inflow velocity. Due to this pulsatile, the maximum flow speed in the microvessel (Ø40 μm [56]) rose up to 2.13 cm/s. The MB is typically filled with gas (e.g., perfluoropropane gas) in the biocompatible shell (e.g., protein) [57]. Thus, the surface tension of MB was set as 0.1 N/m. In addition, Wrede et al. have demonstrated that the ratio (τ) between shell thickness and MB radius affects the ARF direction [6]. When τ < 0.10, the acoustic contrast factor (ACF) of MB is negative, and MB would move towards the high-pressure region (i.e., pressure anti-node). Conversely, MB would move towards the low-pressure regions (i.e., pressure node). In our simulation, MB radius was set to 10 μm, and the shell thickness was 50 nm [57]. Further, the mean density (ρ_mb_) of hollow MB can be calculated as [6]:

ρ_mb_ = ρ_shell_ × (1 − (1 − τ)^3^) (8)

where ρ_shell_ is 1050 kg/m^3^ [58].

## 3. Results and Discussion

Our work presents a machine learning-based model to modulate phase and amplitude patterns of elements capable of rapidly generating an acoustic trap at the predefined target. The model was trained separately using datasets collected at four sample densities, assessing the impact of sample size on model prediction. The resulting performance was analyzed, with details described in the following section. A threshold for sample density (*D*) was identified, which trades off the prediction accuracy against the computational expense. At this density, the accurate ToF prediction ensures trap generation at given targets with different penetration depths. Then, the predicted amplitudes were used to modulate the elements’ emission pressure, updating the trap’s acoustic intensity profiles.

### 3.1. ToF Prediction

**Figure 4:**
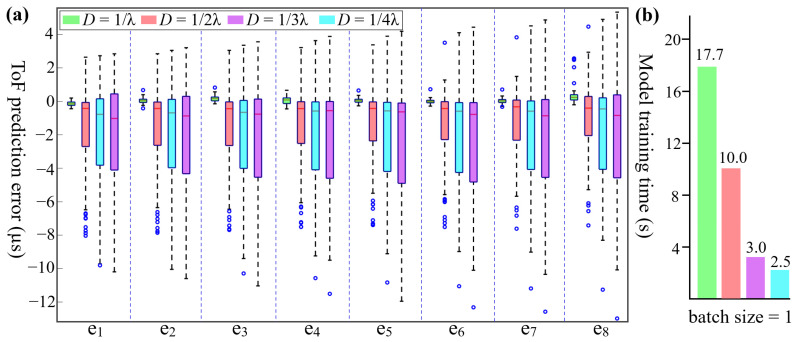
ToF prediction performance of the model trained separately using datasets with four sample sizes. (**a**) Prediction errors of eight elements (i.e., e_1_, e_2_, …, e_8_). The higher sample density (*D*) corresponds to the larger sample size. When *D* = 1/λ, the errors remain range from −0.45 µs to 0.67 µs, with few outliers. Below this density, the errors surge, and many outliers occur. (**b**) Average model training time over 10 runs using datasets with four sample sizes.

ToF is the most significant parameter, so its prediction accuracy directly determines the trap generation. Considering that the accuracy of the learning-based model is generally affected by the sample size [59], a large number of samples are expected for our model training, but this concurrently demands excessive computing resources to collect samples. Specifically, each FE simulation run, which employs massive geometric meshes to discretize the 2D MR anatomy, takes several hours to collect one sample (about 75 min in our study). Note that the sampling method itself is not the core focus of our work. Both FE modeling and the aforementioned MR-ARFI can serve as sampling techniques. Our primary concern on dataset collection is to identify an optimal sample density to balance the prediction accuracy and sample size. To date, the sample density for learning-empowered trap generation has yet to be delved into. Inspired by the sampling strategy in our previous learning-based FUS work [54], we initiated the dataset collection at a sample density of *D* = 1/λ. A total of 920 samples were collected, with 115 samples for hold-out testing. Out of the remaining samples, three subsets consisting of 244, 122, and 65 training samples were selected, corresponding to sparser sample densities of 1/2λ, 1/3λ, and 1/4λ, respectively. These four datasets were used for model training separately. In Figure 4a, at densities of 1/λ, except for a few outliers (only 11 outliers out of 920 predictions), the errors remain within −0.45 µs to 0.67 µs, demonstrating high prediction accuracy. When reducing densities, the maximum errors increased notably by approximately tenfold. Moreover, the presence of numerous outliers indicates large fluctuations in accuracy. Thus, it is evident that a larger sample size indeed raises our model’s prediction accuracy. The prediction efficacy based on these four groups will be validated via FE modeling in later sections (see Section 3.2 and Section 3.3 for details).

In addition to accuracy, model training time also needs to be evaluated. In future clinical practice, the training process must be completed quickly once the data collection is finished using MR-ARFI. This is because patients cannot remain still for long periods to avoid position shifts between the array and body. In the training process, a batch size of 1 was used for the above four training datasets. Figure 4b shows the average model training times over 10 runs, which were 17.7 s, 10.0 s, 3.0 s, and 2.5 s, respectively. This indicates that the proposed model for ToF prediction can deliver rapid model training, offering significant potential for clinical practice.

### 3.2. Phase-Only Modulation for Focal Beam

Since correct PM can ensure trap generation [10,43], we used the predicted ToF to compute phase patterns and input them into FE modeling to visualize the acoustic field. This approach can examine if the ToF accuracy is adequate for trap generation at the target. Given the widespread application of single-beam traps to concentrate MBs [6], we initially validated the generation of a focal beam via PM. This modulation used eight elements, each with a representative diameter of 3.7 mm, referring to the custom-made phased arrays listed in Table 1. This kind of small element is increasingly preferred for recent lab-based research, attributed to their flexible sonication delivery, improved trapping stiffness, and high trapping resolution [18,41]. In contrast, commercial transducers usually have larger diameters due to manufacturing processes, production costs, etc. To investigate our model’s adaptability for the commercial transducer, PM was repeatedly performed using eight elements with a diameter of 7 mm, paralleling the specifications of transducer PK4GA7P1 (Thorlabs Inc., Newton, NJ, USA).

**Figure 5:**
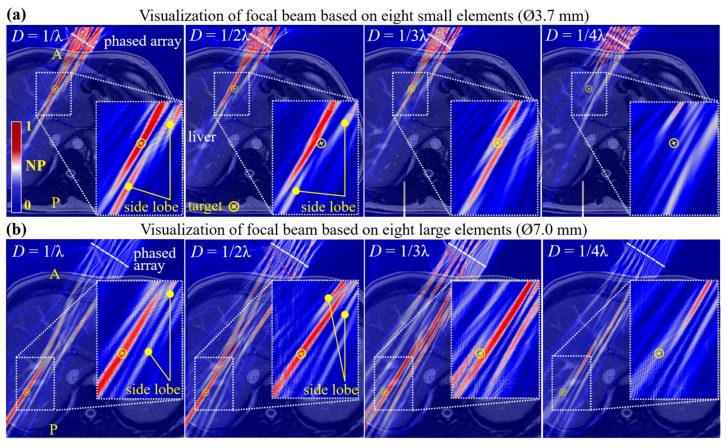
Visualization of focal beam based on two phased arrays with different apertures. The size of the (**a**) small elements is 3.7 mm, and the (**b**) large element is 7.0 mm. When *D* was not less than 1/λ, both kinds of phased arrays formed a focal beam at the given position. Letters “A” and “P” in yellow color indicate the anterior and posterior abdominal walls, respectively.

Figure 5a shows four acoustic fields formed on the same 2D abdominal anatomy. The warmer colors indicate the higher pressures. At sample densities of 1/λ, a finger-like cylindrical region was successfully generated along the propagation direction and covered the target position, signifying a focal beam. At densities of 1/2λ, the focal beam was still generated but drifted from the target position, indicating that ToF prediction accuracy becomes unreliable. Notably, the unwanted side lobes occur in both focal beams, which potentially induce unnecessary tissue injury [54]. Unlike the heat deposition within the focal spot of FUS, the acoustic energy of a focal beam distributes throughout a finger-like region, effectively moderating the temperature rise. Thus, the side effects of lobes in trapping applications are not as severe as FUS. An *in vivo* study on acoustic traps used low-duty cycle (e.g., 1–10%) actuation signals to regulate the energy delivery, showing commendable safety [14]. As the density reduces to 1/3λ, two off-target focal beams emerge, and at 1/4λ, the focal beam fully disappears. Figure 5b shows four acoustic fields using eight larger elements (Ø7 mm). Another representative point, with deeper penetration depth inside the liver, was predefined as the new target. Consistent with the trends observed in Figure 5a, PM again enabled an accurate generation of the focal beam, as sample density was not below 1/λ. Moreover, the side lobes were also observed. Given the above comparison based on different element sizes and penetration depths, the threshold (1/λ) can be a precedent for dataset collection in subsequent studies, reaching a balance between the prediction accuracy and sample size. In addition, the results demonstrate that our model can adapt to both custom-made and commercial elements, indicating its practical applicability.

### 3.3. Phase-Only Modulation for Twin Trap

**Figure 6:**
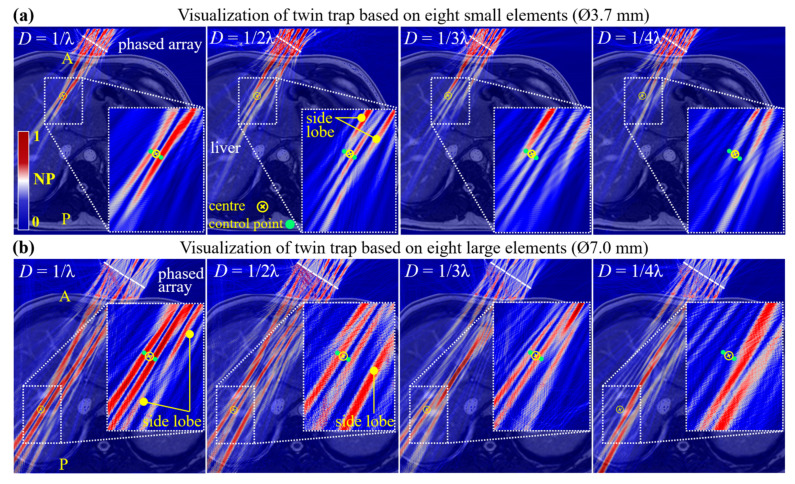
Visualization of twin traps based on two-phased arrays with different apertures. The size of the (**a**) small elements was 3.7 mm, and the (**b**) large elements was 7.0 mm. When *D* was not less than 1/λ, both arrays formed twin traps at the given positions. A pair of solid green circles represented twin trap’s two control points.

Apart from the focal beam acting as a trap, vortex-, bottle-, and twin-shaped acoustic structures also function effectively as acoustic traps. Based on HAEF, these traps can be transformed from a focal beam [10]. Among them, the vortex trap has been successfully applied to concentrate MBs within a mouse’s microvessels [14]. Considering the stronger pulsatility and larger tumor volume in humans, our work focuses on a twin trap, which offers superior lateral force and a bigger working volume compared to a vortex trap [10,15]. A twin trap is interpreted as the combination of two focal beams generated at two designated control points, typically spaced 2λ apart (Figure 6a) [11]. This acoustic structure suggests that MBs with negative ACF will be aggregated in the two focal beams (see Section 3.6 for details). Specifically, MBs will be confined to form two clusters within two focal beams. Once the clusters attain a certain size, the Stokes force they experience exceeds ARF, and they escape the focal beams and then disintegrate into smaller clusters or individual MB [16]. We believe that two microbubble seeding sites created by the twin trap around the tumor potentially increase both the absorption rate and speed compared to a single seeding site facilitated by a single-beam trap. Therefore, in this section, we engage in exploring the generation of twin traps via PM. All acoustic fields were visualized via FE modeling, taking into account three factors: sample sizes, element sizes, and penetration depth.

In Figure 6a, at sample densities of 1/λ, two finger-like cylindrical regions signify high-pressure zones, which shape a twin trap with the low-pressure region in between. Notably, at 1/λ, despite that the twin-shaped acoustic structure is complete, the pronounced amplitude difference between the two focal beams is evident. This phenomenon has not been presented in a single medium [11,15]. It suggests that tissues’ heterogeneity leads to a non-uniform wavefront, thereby making elements contribute unequal pressure to the dual focal beams. As the density decreased, two finger-like regions around the target contracted substantially and eventually vanished. The fields depicted in Figure 6b reaffirm our model’s efficacy for large-sized transducers and deeper penetration. The sample density of 1λ remained a reliable threshold for twin trap generation. At 1/2λ and 1/3λ, while two focal beams still persisted, they increasingly deviated from the target position, and the acoustic intensity of side lobes turned stronger than the twin trap. Note that these two elongated finger-like focal beams, which almost traversed the liver, will not block red blood cells, thus not causing potential hemolytic lysis. The previous experiment has proven that the ARF exerted on red blood cells is far less than the forces exerted on MBs [16].

### 3.4. Phase–Amplitude Modulation

Previous works predominantly focused on PA [9,10,11], with limited effort dedicated to AM. Preliminary attempts have been made to alter the trap’s acoustic intensity profile by equally scaling elements’ emission pressure in a single-medium environment [40]. This method is easy, as it does not require prior knowledge of wave attenuation in beam transmission channels. However, in heterogeneous media, such simplistic AM becomes inapplicable due to different attenuation across all channels. The works in FUS successfully realized an amplitude compensation for attenuating effects and recreated a uniform acoustic wavefront [43,44]. This AM is acknowledged for reducing sidelobes and refining the quality of the focus [44]. Drawing inspiration from these works, we applied this AM strategy in our learning-empowered traps.

**Figure 7:**
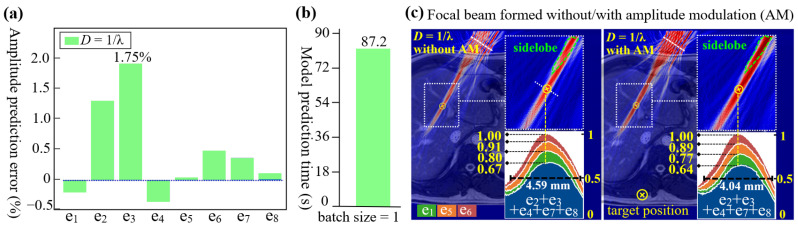
Performance evaluation on focal beam formed without and with AM. (**a**) Average prediction errors of eight elements at the validated sample densities (i.e., 1/λ). Their maximum errors were less than 1.75%. (**b**) Average model training time over 10 runs. (**c**) Comparison of two focal beams formed without and with AM. The left one formed without AM served as a baseline. Two stacked figures showed the normalized pressure changes at target’s lateral direction across a 3.5λ span, as elements were activated in sequential order. The bottom blue layer represents the beam profile formed when five elements (i.e., e_2_, e_3_, e_4_, e_7_, and e_8_) were simultaneously activated. The turquoise, orange, and red layers represent the beam profiles of e_1_, e_5_, and e_6_, respectively. Their full-width-at-half-maximum of the acoustic intensity profile at the target was 4.59 mm and 4.04 mm, respectively.

Figure 7a shows that the average errors of 115 runs of amplitude prediction stay below ±2% at 1/λ. This indicates the training dataset, collected at or above threshold density (i.e., *D* ≥ 1/λ), ensures high precision for amplitude prediction. Note that the prediction has a few outliers, which potentially lead to inaccurate amplitude modulation on certain element(s). The cases for three sparser sample densities (i.e., 1/2λ, 1/3λ, and 1/4λ) were not further discussed. Figure 7b shows the average training time over 10 runs. Along with the above model training for phase prediction, our model can train two ANNs within 2 min. Figure 7c illustrates the focal beams formed without and with AM at the same representative target, where the phase-only modulation served as a baseline. Two focal beams exhibited notable differences, wherein the latter showed warmer colors. We plotted their beam profiles in a lateral direction across a 3.5λ span. The stacked figures reflected changes in normalized pressure when three randomly selected representative elements (i.e., e_1_, e_5_, and e_6_) were activated sequentially alongside the constant activation of e_2_, e_3_, e_4_, e_7_, and e_8_. This method can verify if the AM can recreate a uniform wavefront. In the baseline, the increases in normalized pressure were different: 0.13 by e_1_, 0.11 by e_5_, and 0.09 by e_6_. In comparison, AM made the increases uniform, all increases (i.e., 0.13, 0.12, 0.11) closely approximated the ideal value of 0.125 (=1/8). These values proved that AM could create a uniform wavefront in heterogeneous media. As a result, their full-width-at-half-maximum of the acoustic intensity profile at the target decreased from 4.59 mm to 4.04 mm. This improvement indicates a higher focusing quality, which is consistent with the results observed in FUS [44]. However, we did not find a reduction in sidelobes. The possible reason may be attributed to the different arrangement of elements, where the FUS uses elements arranged on a spherical surface [44,54]. Regarding the twin trap, each element contributed different pressure to two control points rather than to a single focal spot. Therefore, while the current AM can still be used to change the twin trap’s acoustic intensity profile (as shown in Figure 2), it is unable to recreate two uniform wavefronts at both control points.

### 3.5. Computation Efficiency

Since the medical potential of acoustic traps necessitates prompt phase–amplitude modulation, our model’s computational efficiency needs to be evaluated. Among the existing approaches, IB currently cannot solve complicated acoustic fields in thick tissues [14,42]. In addition, HAEF has low accuracy in heterogeneous media and needs a hydrophone to calibrate the phase, making it unsuitable for non-invasive scenarios [40]. Therefore, we compared the computation efficiency between the proposed learning-based model and TRM. The time for 10 runs was averaged for evaluation, and each run included 115 testing samples. Note that we only compared the model trained using the dataset collected at a sampling density of *D* = 1/λ. The TRM computation was conducted in COMSOL, and its maximum mesh size was set at 1/8 wavelength. The scripts of the TRM-based model were run on a workstation (AMD 3990X, 32 GB RAM) without using GPU acceleration. As shown in Table 2, the machine learning-based model is much more efficient in computation, differing by four orders of magnitude compared to TRM. Our model can update the phase–amplitude modulation in real-time (<10 ms), indicating the potential of acoustic traps in future clinical practice.

### 3.6. Microbubble Trapping Capacity

**Figure 8:**
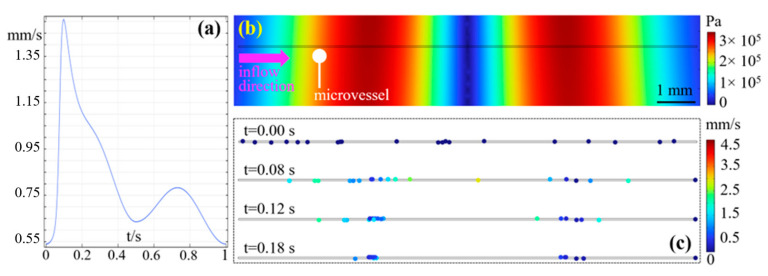
Numerical simulation on the microbubble trapping in pulsatile flow. (**a**) Curve of inflow velocity. Its period is 1 s, and the maximum velocity is up to 1.54 mm/s. It was used to drive the fluid inside microvessel. (**b**) Acoustic field pattern of the twin traps around the predefined microvessel. The maximum pressure is about 340 KPa. The microvessel diameter is 40 μm, and the fluid enters from the left side and flows to the right. (**c**) MB transient distribution at t = 0 s, 0.08 s, 0.12 s, and 0.18 s in COMSOL simulation. At t = 0 s, all MBs do not experience ARF and move due to fluid dynamics. After triggering the twin trap, the MBs (ACF < 0) gradually accumulated in two highest pressure spots.

Focal beam has been validated to be capable of trapping hollow MBs with negative ACF at the pressure anti-nodes [6]. Regarding twin trap, the current works only demonstrated it can trap solid particles (ACF > 0) between two focal beams [10,11]. However, the effect on hollow MB with negative ACF has not been investigated. In this section, FE modeling was built to display the process of MB trapping using a twin trap. The trajectory of each MB was shown dynamically. As shown in Figure 8a, it shows the curve of inflow velocity. Its period is 1 s, which was applied to the inlet of microvessel. In the straight microvessel (Ø40 μm), the flow speed can rise to 2.13 cm/s (Video S1, see Supplementary Materials), matching with the mean velocity of blood flow in capillary [16]. In Figure 8b, it shows the part of acoustic field pattern around the microvessel. Its maximum pressure is about 340 KPa, which is a typical acoustic pressure level in *in vivo* study [55]. After the twin trap was generated, the MBs experienced ARF. As shown in Figure 8c, at four different time points (i.e., 0 s, 0.08 s, 0.12 s, and 0.18 s), the MBs gradually moved towards adjacent pressure anti-nodes. Note that the MB diameter in Figure 8c was enlarged by a scale factor of 12 after completing the computation process only for clear demonstration. This result proves that twin trap can accumulate MBs (ACF < 0) in the encounter flow condition (Video S2, see Supplementary Materials). Compared with the single accumulation spot produced by focal beam, the twin trap can provide two spots for MB accumulation.

## 4. Conclusions

In this study, we propose a pre-concept that uses machine learning to rapidly generate acoustic traps in 2D abdominal anatomy. It aims to advance the trap’s medical potential of concentrating drug carriers (e.g., microbubbles) in pulsatile flows. Accredited to the advances in MRI for sensing acoustic interference, the acoustic trap would be visualized using MRI. In our work, we currently use FE modeling to visualize the trap’s acoustic field pattern. By mapping from trap position to array actuation signals, two ANNs can be trained rapidly (<2 min). This would reduce the patient waiting time and prevent relative movement between body and array in future medical scenarios. After model training, the results suggest that a sampling density of D = 1/λ can act as a reliable reference to balance the sampling time and prediction accuracy. The achieved high ToF prediction accuracy, with error margins ranging from –0.45 µs to 0.67 µs (with only a few isolated outliers), will ensure that the trap position aligns with the predefined microvessel. Meanwhile, the real-time MRI-guided MB tracking will provide visual feedback to surgeons in future medical scenarios, allowing surgeons to select a more suitable microvessel on-site. Our model’s high efficiency in prediction satisfies the rapid adjustment to the trap position. It delivers fast updates to phase modulation in real-time. Such computation efficiency far surpasses that of HAEF and TRM. The predicted amplitude can be used to modulate the transducers’ emission pressure, thereby optimizing the acoustic intensity profiles of traps. The AM results demonstrated an improvement in the focusing quality of the single-beam trap but did not show a reduction in the sidelobes. Furthermore, the trap generation has been successfully replicated when changing the transducer sizes and target penetration depth, which indicates our model’s applicability for future clinical practice.

Our study demonstrates that machine learning can deliver accurate and rapid prediction for ToF and amplitude, enabling the modulation of acoustic trap patterns. However, this pioneering work is only realized in 2D anatomy, and it currently falls short in real clinical applicability. Future efforts in acoustic traps will focus on 3D media, including the dataset collection and model training in 3D, to enhance its practical operability. In the data collection process, we currently use MR anatomy to simulate heterogeneous media, which is insufficient to reflect the complexity of human tissues. Such datasets will weaken the model’s accuracy and reliability. MR-ARFI is likely to be a promising method for data collection. It would visualize the trap’s acoustic field and measure its amplitude in phantoms or the human body so that the mapping between traps and array elements (i.e., phase and amplitude) can be created without requiring prior knowledge of tissue structures. Moreover, MR-ARFI can also consider human physiological activities (e.g., respiration), thereby enriching the dataset’s diversity to improve the trained model’s robustness.

## Figures and Tables

**Table 1:** Major parameters of single-side phased array in previous works.

Literature	Frequency	Wavelength *	Element Diameter	Element Gap	Traversed Media
	[MHz]	[mm]	[mm]	[mm]	
Kang et al. (2010) [53]	1.00	1.50	5.08 × 5.08	0.51	Water
Ghanem et al. (2020) [34]	1.50	1.00	7.0	0.50	Pig bladder, water
Hu et al. (2021) [11]	1.04	1.44	2.6 × 2.6	0.20	Water
Yang et al. (2022) [43]	1.04	1.44	2.6 × 2.6	0.20	Macaque skull, water

* denotes the acoustic speed in water.

**Table 2:** Computation efficiency comparison.

Approach	Average Time for ToF	Average Time for Amplitude
	[ms]	[ms]
Learning-based model	3.5	5.7
TRM	1.5 × e5	

## Data Availability

Our designed files are accessible on the GitHub repository (https://github.com/mengjwu/acoustictrap, accessed on 6 September 2024), such as FE modeling files, anatomy models, and Python codes.

## Supplementary Materials

The following are available online at https://www.mdpi.com/article/10.3390/s24196342/s1, Video S1: Simulated fluid flow velocity in a microvessel (Ø40 μm) using COMSOL. Video S2: Microbubbles (with negative ACF) trapping process in a microvessel (Ø40 μm) via a twin trap.
